# Supplementary material for: Content-rich biological network constructed by mining PubMed abstracts
Source: BMC Bioinformatics. 2004 Oct 8;5:147. doi: 10.1186/1471-2105-5-147 (PMC528731; doi:10.1186/1471-2105-5-147)
Supplement: Additional File 2 — The original results of the above study (non-essential files are deleted to keep the file size under the limit set by BMC bioinformatics). [file 1471-2105-5-147-S2.bz2 › chilibotAdditionalFile2/dip30/761ID8917517E18148/html/DNAB_DNAG.html]

 


 **DNAB** and **DNAG** 
  
Found 91 abstracts in PubMed, retrieved 30.  
 

 What does Google say? 
 PDF only 
| .edu only 

---

**Interactive relationship** (e.g. stimulation, inhibition, etc)

**Neutral relationship**- Mapping protein protein interactions within a stable complex of DNA primase  [ **DNAG** ]  and  **DnaB**  helicase from Bacillus stearothermophilus.  Ref: 10625492 Biochemistry, 2000
- Identification of a region of Escherichia coli  **DnaB**  required for functional interaction with  **DnaG**  at the replication fork.  Ref: 10833513 J Biol Chem, 2000
- We have mapped the surfaces of interaction on both  **DnaG**  and  **DnaB**  and show further that mutations that reduce the physical interation also cause a significant reduction in primer synthesis.  Ref: 8917517 Proc Natl Acad Sci U S A, 1996
- We have shown previously that a protein protein interaction between  **DnaG**  and  **DnaB**  is required to attract the primase to the replication fork.  Ref: 8702920 J Biol Chem, 1996
- Thus, the interaction between  **DnaG**  and  **DnaB**  at the replication fork is the primary regulator of the cycle of Okazaki fragment synthesis.  Ref: 8702921 J Biol Chem, 1996
- Mutations in this region of  **DnaB**  affect the  **DnaB**   **DnaG**  interaction during both general priming directed and phiX174 complementary strand DNA synthesis, as well as at replication forks reconstituted in rolling circle DNA replication reactions.  Ref: 10833513 J Biol Chem, 2000
- Direct physical interaction between  **DnaG**  primase and  **DnaB**  helicase of Escherichia coli is necessary for optimal synthesis of primer RNA.  Ref: 8917517 Proc Natl Acad Sci U S A, 1996
- The primase  **DnaG**  of Escherichia coli requires the participation of the replicative helicase  **DnaB**  for optimal synthesis of primer RNA for lagging strand replication.  Ref: 8917517 Proc Natl Acad Sci U S A, 1996
- Initiation of SPP1 replication is independent of the host DnaA replisome organizer,  **DnaB** , DnaC and DnaI products, but requires the  **DnaG**  DNA primase  [ **DNAG** ]  and the DNA gyrase.  Ref: 8126723 J Mol Biol, 1994
- As we have demonstrated previously,  **DnaG**  is recruited to the replication fork via a transient protein protein interaction with  **DnaB** .  Ref: 10833513 J Biol Chem, 2000
- ... seven proteins, PriA, PriB, PriC, DnaT,  **DnaB** , DnaC, and  **DnaG** , were required for primosome assembly on phi X174 DNA, only  **DnaB** , DnaC, and  **DnaG**  were required for replication from oriC, suggesting that the other proteins were not involved in chromosomal replication.  Ref: 10506828 Prog Nucleic Acid Res Mol Biol, 1999
- Homogenous assays for Escherichia coli  **DnaB**  stimulated  **DnaG**  primase and  **DnaB**  helicase and their use in screening for chemical inhibitors.  Ref: 12009693 Anal Biochem, 2002

**Non-interactive relationship** (e.g. studied together, co-existance, homology, etc.)

- The initiation proteins of the host,  **DnaB**  and  **DnaG** , and the chaperones DnaJ and DnaK are not required for P4 replication, because polyclonal antibodies against those polypeptides do not inhibit the process.  Ref: 8029013 Nucleic Acids Res, 1994
- RepA protein of pPS10 promotes in vitro plasmid replication in extracts from E. coli, and this replication depends on host proteins DnaA,  **DnaB** ,  **DnaG** , and SSB.  Ref: 7635822 J Bacteriol, 1995
- The  **DnaB**  homolog from Arabidopsis also contains a  **DnaG**  primase domain and the  **DnaB**  homolog from the nematode seems to contain an inactivated version of the primase.  Ref: 10645945 Genome Res, 2000
- There are two types of such systems, the first one represented by distinct helicase and primase proteins e.g.,  **DnaB**  and  **DnaG**  proteins of Escherichia coli, and the second one by single polypeptides comprising both activities gp4 of bacteriophages T7 and T3, and alpha protein of bacteriophage P4.  Ref: 1569588 J Mol Evol, 1992
- Using a combination of biochemical and physical techniques, we show that the helicase primase complex comprises one  **DnaB**  hexamer and either two or three molecules of  **DnaG** .  Ref: 10625492 Biochemistry, 2000
- The primosome is a mobile multiprotein DNA replication priming apparatus that requires seven Escherichia coli proteins replication factor Y protein n, proteins n and n, and the products of the  **dnaB** , dnaC, dnaT, and  **dnaG**  genes for assembly at a specific site termed a primosome assembly site on single stranded DNA binding protein coated single stranded DNA.  Ref: 2547799 J Biol Chem, 1989
- iii Mutations in either the dnaA,  **dnaB** , dnaC, dnaE,  **dnaG** , or ssb gene, when introduced into a strain containing a bioA lac fusion, produced a blue colony color without an increase in beta galactosidase synthesis.  Ref: 2142938 J Bacteriol, 1990
- The multiprotein primosome which includes the  **DnaB**  and  **DnaG**  proteins, defined biochemically on the basis of its requirement during bacteriophage phi X174 complementary strand synthesis, could serve as the helicase primase replication machine on the lagging strand template.  Ref: 1938875 J Bacteriol, 1991
- Kid inhibits in vitro replication of CoEl to a basal level without altering the superhelicity of the template but it does not substantially affect in vitro replication of P4, a DnaA,  **DnaB** , DnaC and  **DnaG**  independent replicon.  Ref: 7723014 J Mol Biol, 1995
- At non permissive temperature, plasmid replication depends on the  **dnaB** , dnaC,  **dnaG**  and dnaI initiation replication products, but does not require the dnaA function.  Ref: 8339905 FEMS Microbiol Lett, 1993
- The replication restart proteins, PriA, PriB, PriC,  **DnaB** , DnaC,  **DnaG** , DnaT and Rep, form redundant pathways that recognize repaired replication forks and restart them.  Ref: 11532137 Mol Microbiol, 2001
- For the first time, we demonstrate directly a stable complex between a bacterial  **DnaG**  primase and  **DnaB**  helicase.  Ref: 10625492 Biochemistry, 2000
- The fundamental activities of the replicative primosomes of Escherichia coli are provided by  **DnaB** , the replication fork DNA helicase, and  **DnaG** , the Okazaki fragment primase.  Ref: 10833513 J Biol Chem, 2000
- These include some of the subunits of the DNA polymerase III holoenzyme, the  **DnaB**  replication fork helicase, and the  **DnaG**  primase.  Ref: 1938875 J Bacteriol, 1991
- In Escherichia coli, the primosome assembly proteins, PriA, PriB, PriC, DnaT, DnaC,  **DnaB** , and  **DnaG** , are thought to help to restart DNA replication forks at recombinational intermediates.  Ref: 10835375 Genetics, 2000
- the alpha 3 mutant has an additional replication origin whose function is dependent on  **DnaB**  and DnaC proteins, rather than on  **DnaG**  protein alone.  Ref: 2325622 Mol Gen Genet, 1990
- However, recombination was stimulated by mutations in genes encoding replication proteins, including the primosomal proteins  **DnaB** , DnaD and the  **DnaG**  primase, the putative DNA polymerase III subunits PolC, DnaN and DnaX, as well as the DNA polymerase DnaE.  Ref: 11251841 Mol Microbiol, 2001
- In addition, a multicopy  **dnaB**  recombinant, but not a multicopy  **dnaG**  recombinant, prevents the toxicity associated with this protein.  Ref: 7723014 J Mol Biol, 1995
- Gel filtration chromatography was used to isolate both preprimosomal and primosomal complexes formed on single stranded DNA binding protein coated phiX174 DNA by the combination of PriA, PriB, PriC, DnaT,  **DnaB** , DnaC, and  **DnaG** .  Ref: 8663105 J Biol Chem, 1996
- This can be attributed to its ability, unlike wild type DnaC, to catalyze a SSB insensitive general priming reaction with  **DnaB**  and  **DnaG**  on any SSB coated single stranded DNA.  Ref: 10713144 J Biol Chem, 2000
- The behavior of the purified mutant  **DnaB**  proteins in the various replication systems suggests that access to the  **DnaG**  binding pocket on  **DnaB**  may be restricted at the replication fork.  Ref: 10833513 J Biol Chem, 2000
- Bacteriophage Mu A protein, which remains tightly bound to the Mu ends in the native strand transfer intermediate, blocked initiation of Mu DNA replication by a system of 8 host proteins  **DnaB**  helicase, DnaC protein,  **DnaG**  primase, DNA polymerase III holoenzyme, DNA polymerase I, DNA gyrase, DNA ligase, and single strand binding protein.  Ref: 8206956 J Biol Chem, 1994
- However, previous studies had not determined whether the activation of the primase or its loading on the template was accomplished by a helicase mediated structural alteration of the single stranded DNA or by a direct physical interaction between the  **DnaB**  and the  **DnaG**  proteins.  Ref: 8917517 Proc Natl Acad Sci U S A, 1996
- The bacterial RNA polymerase, the initiation protein DnaA, and the primosomal proteins  **DnaB** , DnaC,  **DnaG**  and DnaT are not required.  Ref: 1851552 Nucleic Acids Res, 1991
- The initiation of replication from oriV RSF1010, the replication origin of the broad host range plasmid RSF1010, depends on RepA helicase, RepB primase, and RepC initiator protein, encoded by RSF1010 itself, while this initiation event in E. coli is independent of dnaA,  **dnaB** , dnaC, and  **dnaG**  Scherzinger et al.  Ref: 8690079 FEBS Lett, 1996
- The assay uses an adaptation of the general priming reaction by employing  **DnaG**  primase,  **DnaB**  helicase, and ribonucleotidetriphosphates incorporation of 3 H CTP for in vitro primer synthesis on single stranded oligonucleotide and M13mp18 DNA templates.  Ref: 12009693 Anal Biochem, 2002
